# Utilization of Reproductive Health Services and Associated Factors among Secondary School Students in Woldia Town, Northeast Ethiopia

**DOI:** 10.1155/2021/2917874

**Published:** 2021-04-20

**Authors:** Teshome Gebremeskel Aragie, Biruk Beletew Abate

**Affiliations:** Woldia University College of Health Sciences, Department of Medical Anatomy, P.O. Box 400, Amhara, Ethiopia

## Abstract

**Background:**

Reproductive health is a universal concern but it has special importance for women particularly during the reproductive year. Although policy actions and strategic efforts made reproductive health service uptake of youths in Ethiopia, still its utilization remains low. Adolescence is not quite capable of understanding complex concepts. This makes them vulnerable to sexual exploitation and high-risk sexual behaviors and reproductive health problems.

**Objective:**

The aim of this study was to assess the utilization of reproductive health services and associated factors among adolescents in Woldia town secondary schools, Amhara, Ethiopia, 2019.

**Methods:**

An institutional-based descriptive cross-sectional study was conducted on 420 secondary school students in Woldia Town from January to June 2019. A self-administered, structured questionnaire was used to collect the data. The samples were distributed proportionally, and participants in each school were selected by the systematic sampling technique. Bivariable and multivariable logistic regression was carried out to assess the association between dependent and independent variables.

**Result:**

Out of 420 students participated in this study, 270 (64.3%) of the respondents utilize reproductive health service. Residence (AOR = 4.40, 95%CI (1.23, 9.362)), educational status of the partner (AOR = 2.66, 95%CI (2.35, 5.24)), presence of RHS facility in school (AOR = 2.53, 95%CI (1.57, 4.06)), and good knowledge level on reproductive health services (AOR = 1.77, 95%CI (1.14, 2.75)) were significantly associated with reproductive health service utilization.

**Conclusion:**

*and Recommendations*. Knowledge of respondents on reproductive health utilization in the study area was found to be low. Students who were from rural families have low utilization of reproductive health services. This low service utilization in these students might be disposed to different reproductive health risks such as sexually transmitted infections, HIV/AIDS, and unwanted pregnancy, which in turn can increase the school dropout rate and have an impact on an individual's future life. However, students who have good knowledge and were encouraged by their friends have good reproductive health service utilization. Therefore, it needs a great effort and attention of all concerned bodies including parents, school staff, and health professionals to improve service utilization in schools.

## 1. Introduction

According to the World Health Organization (WHO), reproductive health (RH) is defined as a state of complete physical, mental, and social wellbeing and not merely the absence of disease or infirmity in all matters related to the reproductive system and its functions and process [1].

Reproductive health is a universal concern but it has special importance for women particularly during the reproductive year [2]. It addresses the human sexuality and reproductive processes at all stages of life and implies that people can have “a responsible, satisfying and safe sex life” and that they can reproduce and have the freedom to decide when and how often they can do so. Youth and adolescents are characterized by unique physical, psychological, social, and emotional changes that put their life at high risk [3].

The term Youth Friendly Reproductive Health Service (YFRHS) refers to those services that are accessible, acceptable, and appropriate for youths such as counseling, family planning, voluntary counseling and testing (VCT), and treatment of sexually transmitted infections [4]. The services are provided in line with the minimum health package and aim to increase the acceptability and use of health services by young people [5]. Currently, there is a low level of access to high-quality RH information and services, especially for adolescents [6]. In the past few years, the issues of RH have been increasingly perceived as a social problem; they have been emerging as a topic of increasing concern in both developed and developing countries [7].

Adolescence is not quite capable of understanding complex concepts. This makes them vulnerable to sexual exploitation and high-risk sexual behaviors and reproductive health problems [8]. Globally, 45 percent of all new HIV infections worldwide are occurring among young people aged 15 to 24 years. Over 500,000 young people are infected with STI per day; approximately 80 million women have unwanted pregnancies every year [9, 10]. HIV/AIDS prevalence data shows that 15- to 24-year-old people have the highest rates of new HIV infection, with adolescent girls being considerably more likely to be infected than adolescent boys [11]. This is because many adolescents are less informed, less experienced, and less comfortable accessing health services for RH than do adults [12]. Moreover, the magnitude of unsafe abortions in young women aged 15–24 reached 45 percent, and the level of comprehensive knowledge of AIDS is being 48% for young women aged 15–24 and 43% for young men aged 15–24 [13].

Young people in high schools are highly vulnerable and at risk of HIV infection due to various reasons such as unprotected casual sex relationships and multiple sexual partners, lack of comprehensive knowledge about HIV/AIDS, sexual and reproductive health, lack of access to HIV services, sexual experimentation, early sexual debut and peer pressure, and other related factors [14].

In Africa, 430,000 young people are infected with HIV per year, 2.6 million young people are living with HIV, teenage pregnancy rates remain high, and maternal mortality is among the leading causes of death for adolescent girls in this region [15]. There are also problems related to SRH services which include accessibility, availability, and quality. Moreover, secondary school students indicated that health providers in most of the HEIs are not trained to respond to the needs of young people [16]. The shortage of youth-friendly health services and counseling poses significant challenges to address SRH issues, including HIV prevention. In Ethiopia, educational institution-based RH services are often limited by restrictive policies, lack of private areas for counseling, and poor links to resources outside the institutions. Since adolescents have unique reproductive health risks of unplanned pregnancies, childbearing, sexually transmitted infections (STIs) including HIV and unsafe abortion [17, 18], and access and utilization of reproductive health services (RHS) are a primary concern for them surrounding the promotion of reproductive health rights [19, 20].

In addition to these, the response to deliver RHS for students has been fragmented, and Ethiopian schools have limited capacity in delivering health-related services for their students. As a result, a significant proportion of students are developing RH-related problems [20], and the available literature in Ethiopia was limited in addressing factors that influence the utilization of RHS among secondary school students. Hence, the aim of the study was to assess utilization and factors affecting RHS among secondary school students in Woldia town, Northeast Ethiopia.

## 2. Methods

### 2.1. Study Setting and Period

This study was conducted in Woldia town from January to June 2019. The town is located in Northeast Ethiopia Amhara regional state under the administration town of North Wollo Zone, located at about 521 km from Addis Ababa, the capital of Ethiopia. The town has nine kebeles and four secondary schools. The study was conducted in all secondary schools, namely, Woldia secondary school, Millennium secondary school, Selam secondary school, and Gubo secondary school.

### 2.2. Study Design

An institution-based descriptive cross-sectional study was employed.

### 2.3. Source of Population

All secondary school students in Woldia town were the source of population.

### 2.4. Study Population

Regular secondary school students of Woldia town were the study population.

### 2.5. Sample Size Determination

The sample size of the study was determined using single proportion population formula as *n* = [(Z*α*/2)^2^^*∗*^ P (1−P)/d^2^)], where *n* is the sample size, *p* is the prevalence of sexual practice <18 years (taken as *53.3%* according to the study on reproductive health service utilization in Mizan Tepi University students, Ethiopia, 2017 [21]), *d* is the margin of error (*d* = 0.05), and *Z* is the value of standard normal distribution at 95% confidence level (*Z* *=* 1.96). By adding a 10% nonresponse rate of 382^*∗*^(10% = 38), the final sample size was 420.

### 2.6. Sampling Techniques and Procedure

In the study area, from each school, sections were selected by using a simple random sampling method. The samples were distributed proportionally based on probability proportional to size (PPS). Participants from the selected section were selected by using a systematic random sampling technique after calculating sampling interval (K) for each section according to students' roll number in the class and the first student was selected by lottery method.

### 2.7. Eligibility Criteria

Regular secondary school students who were attending their classes during the data collection period were included in this study. However, students who were not available during the data collection period and students who were physically and mentally not able to be interviewed were excluded from this study.

### 2.8. Operational Definitions

*Good Utilization*. Those students whose score was at the mean and above the mean score of practice questions were categorized as having good practice while those students whose score was below the mean score were categorized as having poor practice.

*Good Knowledge*. Those students whose score was at the mean and above the mean score of knowledge questions were categorized as knowledgeable while those students whose score was below the mean score were categorized as being under poor knowledge.

*Good Attitude*. Those students whose score was at the mean and above the mean score of attitude questions were categorized as having a good attitude while those students whose score was below the mean score were categorized as having a poor attitude.

### 2.9. Data Collection Tool and Procedure

Data was collected by using a self-administered structured questionnaire containing written consent, sociodemographic data, and reproductive health-related questions which were developed by adapting from different peer-reviewed studies. The questionnaire was first developed in English and translated to the Amharic language by an expert. It was translated back to English by an independent translator to check for consistency. The data was collected by four diploma clinical nurses and supervised by two master's public health students.

### 2.10. Data Quality Control

For ensuring data quality, training about the purpose of the study, the questionnaire in detail, the data collection procedure, the data collection setting, and the rights of study participants was given to data collectors and supervisors. Pretest of the questionnaire was conducted on 5% of the sampled students in Dessie town secondary school a week prior to the actual survey, and necessary modification was done according to the gap identified. Besides, the completeness, accuracy, and consistency of the collected data were conducted by supervisors and principal investigators during the whole data collection process. Finally, the data were entered daily, and missing data were identified.

### 2.11. Data Analysis and Presentation

Data were entered by using EPI-data Version 4.2.0 and were exported to SPSS Version 24 for analysis. Frequencies were calculated to describe the study population in relation to relevant variables. Binary logistic regression analysis was conducted to assess the crude association between dependent and independent variables. Finally, variables that show an association in binary logistic regression analysis and have a *P* value less than or equal to 0.25 were entered into a multivariable logistic regression model to adjust the effects of possible confounders. Finally, the level of significance was approved at a *P*value less than 0.05.

## 3. Results

All 420 respondents complete the questioner with a response rate of 100%. Among these, most of the respondents (304 (72.4%)) were from urban areas. About 204 (48.6%) were females. The majority of the respondents (238 (56.7%)) were within the age group of 15–19 years. Most of the respondents were single (384 (91.4%)), and 231 (55%) of the students have boy/girlfriends (Table 1).

### 3.1. Utilization of Reproductive Health Services

Out of 420 study participants, 270 (64.3%) utilized reproductive health services. About 266 (63.3) respondents utilized VCT while 190 (65%) respondents utilized family planning service. Nearly half of the respondents (225 (53.55%)) obtain the RHS from the hospital. Greater than half (56.7%) of the respondents have communication with their parents related to RH issues. In this study, most of the respondents (249 (59.3%)) prefer to discuss RH issues with their friends (Table 2).

### 3.2. Reproductive Health Service Utilization and Associated Factors

In bivariable logistic regression analysis, age of the respondent, residence, sex of the respondent, educational level of the respondent, educational status of the partner, presence of RHS facility in school, reason for not getting RHS, encouragement by friends to use RHS, chat chewing, drinking alcohol, and good knowledge level on RHS were associated with the reproductive health service utilization. Those variables that have a *P* value less than or equal to 0.25 were entered into a multivariable logistic regression model to adjust for possible confounders.

In multivariate logistic regression analysis, residence, educational level of the respondent, educational status of the partner, presence of RHS facility in school, the reason for not getting RHS, encouragement by friends to use RHS, and good knowledge level on RHS have been found to be significantly associated with the reproductive health service utilization. Accordingly, the odds of the utilization of reproductive health service among respondents from urban residence (AOR = 4.40, 95%CI (1.23, 8.36)) was higher compared to those respondents from rural residences. The odds of the utilization of reproductive health service among those who have RHS facility in school (AOR = 2.53, 95% CI (1.57, 4.06)) was higher compared to those respondents who have not RHS facility in their school. Regarding the grade level of students, the utilization of reproductive health services among grade 10 students was (AOR = 1.15, 95% CI (1.73, 4.80)) higher compared to grade 9 students.

The odds of RHS utilization among respondents whose partners' educational status falls under secondary and above (AOR = 2.66, 95%CI (2.35, 5.24)) was higher compared to those whose partners' educational status is unable to read and write. Respondents whose neighbors feel ashamed were (AOR = .24, 95% CI (.23, .27)) higher compared to those who have no money to get the service. The odds of RHS utilization among participants whose friends encouraged them to use RHS was (AOR = 1.46, 95%CI (1.87, 6.44)) higher compared to those whose friends did not encourage them to use RHS. Respondents who have good knowledge of RHS were (AOR = 1.77, 95%CI (1.14, 2.75)) higher compared to students who have poor knowledge to utilize reproductive health services (Table 3).

## 4. Discussion

The overall utilization of RHS among secondary school youths in Woldia town was found to be 64.3%; the finding was comparable with the study conducted in Nigeria [22] and Harar in which 63.8% of youths utilize reproductive health services [23]. However, it was higher than the study conducted in Jimma [24], Nekemet [25], Madawalabu University [26], Mekele [27], and Mechakel, East Gojjam [8], in which 21.2%, 27.7%, 23%, and 21.5% of participants utilized RHS, respectively. The possible reason for this difference might be due to the participants' sociodemographic characteristics, duration of time reference used in the definition of RHS utilization, and socioeconomic variation. Furthermore, this discrepancy might be due to differences in the availability or accessibility of youth-friendly health facilities or youth centers in the school, educational status/level, socioeconomic status, urban-rural residence, transportation, and cultural variations. The odds of the utilization of reproductive health service among respondents from the urban residence was higher compared to those respondents from rural residences. This finding was supported by other studies in Ethiopia [20, 28]. This might be because the accessibility of RHS in the urban area and the availability of media in urban areas will facilitate the information transmission regarding the benefit of utilizing RHS. The odds of the utilization of reproductive health service among those who have RHS facility in school was higher compared to those respondents who have not RHS facility in the school. This finding was supported by other studies in Ethiopia [20, 28, 29]. This might be due to the fact that those school-based RHS facilities will deliver services like counseling and easily accessing the facility students need timely.

In this study, the odds of RHS utilization among respondents whose partners' educational status falls under secondary and above was higher compared to those whose partners' educational status is unable to read and write. This finding was supported by other studies [29–31]. Those families with higher educational status are more likely to be familiar with RHS-related issues. Respondents whose neighbors feel ashamed were 0.24 times utilize RHS compared to those who have no money to get the service. This finding was supported by other studies [31, 32]. This might be because of the stigma due to the utilization of RHS, which will result in isolation from their peers and society as a whole. To prevent this discrimination, they prefer not to use RHS.

The odds of RHS utilization among respondents whose friends encourage them to use RHS was higher compared to those whose friends did not encourage them to use RHS. Other studies also support this finding [31, 33]. This shows the influence of peer pressure on the utilization of RHS. In this study, respondents who have good knowledge of RHS were in a good stance to utilize reproductive health services compared to nonknowledgeable respondents. A similar finding has been reported in other studies [8, 34–36]. The possible justification for this might be the fact that those respondents with a good level of knowledge regarding RHS will understand the benefit of using RHS and the consequence of not using RHS. In this study, a good attitude regarding RHS was 1.14 times at a higher level to utilize reproductive health services. This finding was comparable with the study conducted by Tegegn et al. in southwest Ethiopia [36].

## 5. Conclusions and Recommendations

Knowledge of respondents on reproductive health utilization in the study area was found to be low. Students who were from rural families have low utilization of reproductive health services. This low service utilization in these students could dispose them to different reproductive health risks such as sexually transmitted infections, HIV/AIDS, and unwanted pregnancy, which in turn can increase the school dropout rate and have an impact on an individual's future life. However, students who have good knowledge and were encouraged by their friends have good reproductive health service utilization. Therefore, special attention is needed for students from rural families regarding their parent-adolescent open communication, school staff involvement in their relationship, and open discussion on their sexual and reproductive health issues, and stakeholders should work to scale up the service utilization in schools by considering the students' background.

## Figures and Tables

**Table 1:** Sociodemographic characteristics of secondary school students in Woldia town Woldia, Amhara, Ethiopia.

Characteristics	Category	Frequency	Percent
Age	10–15	50	11.9
	15–19	238	56.7
	20–24	132	31.4

Sex	Male	216	51.4
	Female	204	48.6

Marital status	Single	384	91.4
	Married	21	5.0
	Divorced	13	3.1
	Widowed	2	.5

Residence	Urban	304	72.4
	Rural	116	27.6

Wealth index	High	108	25.7
	Medium	245	58.3
	Low	67	16.0

Educational level	Grade 9	237	56.4
	Grade 10	183	43.6

Presence of RHS in school	Yes	286	68.1
	No	134	31.9

Handling of RHS providers	Good	192	45.7
	Moderate	177	42.1
	Bad	51	12.1

Missed RHS required	Yes	262	62.4
	No	158	37.6

Reason for not getting RH	Lack of money	156	37.1
	Neighbors felt ashamed	147	35.0
	Service providers refused	77	18.3
	Clinic was closed	40	9.5

Ever heard of YFRHS	Yes	261	62.1
	No	159	37.9

Parents influence not to use RHS	Yes	270	64.3
	No	150	35.7

The stigma attached to utilize RHS	Yes	268	63.8
	No	152	36.2

Cultural and religious influence	Yes	248	59.0
	No	172	41.0

Have a girl/boyfriend	Yes	231	55.0
	No	189	45.0

Had sexual intercourse	Yes	150	35.7
	No	270	64.3

Chew chat	Yes	103	24.5
	No	317	75.5

Drink alcohol	Yes	122	29.0
	No	298	71.0

**Table 2:** Utilization of RHS among secondary school students in Woldia Town, Amhara, Ethiopia.

Variables	Category	Frequency	Percent
Have you ever used any of the different RHS	Yes	335	79.8
	No	85	20.2

Counseling service generally	Yes	217	51.70
	No	203	48.3

VCT	Yes	266	63.3
	No	154	36.7

Management of STI/HIV	Yes	171	40.7
	No	249	59.3

Family planning	Yes	273	65
	No	147	35.0

Place where the RHS obtained	Hospital	225	67.1
	Clinic	49	14.3
	Dispensary	13	3.88
	Pharmacy	43	12.8
	Others	5	1.5

Condom use	Yes	219	52.1
	No	201	47.9

Currently using contraceptive	Yes	173	41.2
	No	247	58.8

History of STI	Yes	181	43.1
	No	239	56.9

Ever been pregnant	Yes	91	44.6
	No	113	55.4

Ever had abortion	Yes	51	25
	No	153	75

Parent-teen communication	Yes	238	56.7
	No	182	43.3

Where abortion was conducted	Public health institution	19	37.2
	Private clinic	24	47.0
	Abortion house	6	11.7
	Ingesting different drugs	2	3.92

With whom you prefer to discuss RH issues	Friends	249	59.3
	Partners	106	25.2
	Siblings	25	6
	Partners	20	4.8
	Professionals	14	3.3
	Others	6	1.4

**Table 3:** Factors associated with the utilization of reproductive health services among secondary school students in Woldia, Amhara, Ethiopia.

Variables	Level of practice (*N* = 420)		Odds ratios		
	Good	Poor			
	270 (64.3)	150 (35.7)	Corollary (95% CI)	AOR (95% CI)	*P* value
*Age*					
10–15	12 (2.9)	38 (9.0%)	0.43 (0.21,.90)	0.47 (.21, 1.03)	0.058
15–19	82 (19.5)	156 (37.1%)	0.71 (0.46, 1.104)	0.69 (.43,1.11)	0.127
20–24	56 (13.3)	76 (18.1%)	1	—	—

*Residence*					
Urban	137 (32.6)	167 (39.8)	4.67 (1.08, 8.57)	4.40 (1.23, 9.362)^*∗*^	0.024
Rural	67 (16.0)	49 (11.7)	1	1	—

*Sex*					
Male	70 (16.7)	146 (34.8)	1	1	—
Female	80 (19.0)	124 (29.5)	1.35 (0.90, 2.01)	1.14 (0.74,1.77)	0.555

*Educational level*					
Grade 9	77 (18.3)	160 (38.1)	1	—	—
Grade 10	73 (17.4)	110 (26.2)	1.38 (0.92, 2.06)	1.15 (1.73, 4.80)^*∗*^	0.045

*Educational status of the partner*					
Unable to read and write	27 (6.4)	69 (16.4)	1	1	
Able to read and write	27 (6.4)	55 (13.1)	1.26 (0.66, 2.38)	0.64 (0.35, 1.17)	0.147
Primary school	26 (6.2)	51 (12.1)	1.30 (0.68, 2.49)	0.69 (0.38, 1.25)	0.221
Secondary and above	70 (16.7)	95 (22.6)	3.88 (1.10, 3.24)	2.66 (2.35, 5.24)^*∗*^	0.012

*Youth-friendly RHS facility in schools*					
Yes	81 (9.3)	205 (48.8)	1	—	—
No	69 (16.4)	65 (15.5)	2.69 (1.76, 4.11)	2.53 (1.57, 4.06)^*∗*^	<0.001

*Reason for not getting RHS*					
Had no money	52 (12.4)	104 (24.8)	1	1	—
Neighbors feel ashamed	23 (5.5)	17 (4.0)	0.34 (0.33, 0.50)	0.24 (0.23, 0.27)^*∗*^	0.032
Service providers refused to side the service	26 (6.2)	51 (12.1)	1.02 (0.57, 1.82)	0.84 (0.44, 1.60)	0.598
The clinic was closed	49 (11.7)	98 (23.3)	1.00 (0.62, 1.61)	0.95 (0.56, 1.62)	0.857

*Did your friends encourage you to use RHS*					
Yes	48 (11.4)	124 (29.5)	1.81 (1.19, 2.74)	**1.46 (1.87, 6.44)^∗^**	**0.005**
No	102 (24.3)	146 (34.8)	1	1	—

*Chat chewing*					
Yes	74 (17.6)	29 (6.9)	1	1	—
No	196 (46.7)	121 (28.8)	1.58 (.97, 2.56)	1.08 (.56,2.06)	0.820

*Drink alcohol*					
Yes	36 (8.6)	86 (20.5)	1	—	—
No	114 (27.1)	184 (43.8)	1.48 (0.94, 2.33)	1.17 (0.63,2.17)	0.621

*Knowledge level*					
Good knowledge on RHS	86 (20.5)	118 (28.1)	1.73 (1.16, 2.59)	**1.77 (1.14, 2.75)^∗^**	**0.011**
Poor knowledge on RHS	64 (15.2)	152 (36.2)	1	1	—

*Attitude level*					
Good attitude	63 (15.0)	119 (28.3)	1.29 (1.72, 3.63)	**1.14 (2.73, 5.80)** ^*∗*^	**0.05**
Poor attitude	87 (20.7)	151 (36.0)	—	—	—

Note: ^*∗*^*P* value <0.05, CI = Confidence Interval, COR = Crude Odds Ratio, AOR = Adjusted Odds Ratio.

## Data Availability

The data are available and can be accessed from the corresponding author when asked with a reasonable inquiry.

## Abbreviations

RH: Reproductive health
RHS: Reproductive health services
STI: Sexually transmitted infections
VCT: Voluntary counseling and testing
YFRHS: Youth-Friendly Reproductive Health Services.

## References

[1] McPherson K. E., Kerr S., McGee E. (2014). The association between social capital and mental health and behavioural problems in children and adolescents: an integrative systematic review. *BMC Psychology*.

[2] Tepi Universtiy (2017). *Assessment of Knowledge, Attitude and Practice towards Reproductive Health Service Among Mizan*.

[3] Aboma Motuma T. S., Kenay A., Motuma (2016). Utilization of youth friendly services and associated factors among youth in Harar town, east Ethiopia: a mixed method study. *BMC Health Services Research*.

[4] Open University of Tanzania (2016). *Adolescents’ Awareness of Youth Friendly Reproductive Health Services In Public Health Facilities: The Case of Ilala Municipality Lutende*.

[5] McGorry P., Bates T., Birchwood M. (2013). Designing youth mental health services for the 21st century: examples from Australia, Ireland and the UK. *British Journal of Psychiatry*.

[6] Ojong I., Akpan M., Alasia M. O., Nlumanze F. (2014). A comparative study ON reproductive health awareness among urban and rural secondary school students IN cross river state, Nigeria. *Journal of Research in Nursing and Midwifery*.

[7] Akhter S. (2007). *Knowledge, Attitudes and Practices on Reproductive Health and Rights of Urban and Rural Women in Bangladesh*.

[8] Abajobir A. A., Seme A. (2014). Reproductive health knowledge and services utilization among rural adolescents in east Gojjam zone, Ethiopia: a community-based cross-sectional study. *BMC Health Services Research*.

[9] Alliance A. Y., Finder P. (2003). *Youth Friendly Sexual and Reproductive Health Services: An Assessment of Facilities*.

[10] Coley R. L., Medeiros B. L., Schindler H. S. (2008). Using sibling differences to estimate effects of parenting on adolescent sexual risk behaviors. *Journal of Adolescent Health*.

[11] UNAIDS (2000). *HIV/AIDS. JUNPo. Ethical Considerations in HIV Preventive Vaccine Research: UNAIDS Guidance Document*.

[12] Tegegn A. (2008). Reproductive health knowledge and attitude among adolescents: a community based study in Jimma Town, Southwest Ethiopia. *Ethiopian Journal of Health Development*.

[13] Evens E., Otieno-Masaba R., Eichleay M. (2014). Post-abortion care services for youth and adult clients in Kenya: a comparison of services, client satisfaction and provider attitudes. *Journal of Biosocial Science*.

[14] Barnabas G., Pegurri E., Selassie H. H., Naamara W., Zemariam S. (2014). The HIV epidemic and prevention response in Tigrai, Ethiopia: a synthesis at sub-national level. *BMC Public Health*.

[15] Group W. B. (2014). *World Development Indicators 2014*.

[16] Liu X., Liang L. L., Liu E. (2012). Science education research in China: challenges and promises. *International Journal of Science Education*.

[17] Ingwersen R. (2001). *Youth Reproductive and Sexual Health in the Developing World*.

[18] Adefuye A. S., Abiona T. C., Balogun J. A., Lukobo-Durrell M. (2009). HIV sexual risk behaviors and perception of risk among college students: implications for planning interventions. *BMC Public Health*.

[19] Braeken D., Rondinelli I. (2012). Sexual and reproductive health needs of young people: matching needs with systems. *International Journal of Gynecology & Obstetrics*.

[20] Nafukho F. M. (2007). The place of E-learning in Africa’s institutions of higher learning. *Higher Education Policy*.

[21] Tepi Universtiy (2017). *Assessment of Knowledge, Attitude and Practice towards Reproductive Health Service Among Mizan Tepi Universtiy Tepi Campus Students, Sheka Zone, South Nations Nationalities and Peoples Regional State*.

[22] Odo A. N., Samuel E. S., Nwagu E. N., Nnamani P. O., Atama C. S. (2018). Sexual and reproductive health services (SRHS) for adolescents in Enugu state, Nigeria: a mixed methods approach. *BMC Health Services Research*.

[23] Motuma A. (2012). Youth-friendly health services utilization and factors in Harar, Ethiopia. *Harar Bulletin of Health Sciences*.

[24] Tegegn A., Gelaw Y. (2009). Adolescent reproductive health services in jimma city: accessibility and utilization. *Ethiopian Journal of Health Sciences*.

[25] Binu W., Marama T., Gerbaba M., Sinaga M. (2018). Sexual and reproductive health services utilization and associated factors among secondary school students in Nekemte town, Ethiopia. *Reproductive Health*.

[26] Dida N., Darega B., Takele A. (2015). Reproductive health services utilization and its associated factors among Madawalabu University students, Southeast Ethiopia: cross-sectional study. *BMC Research Notes*.

[27] Kahsay K., Berhe S., Alemayehu M. (2016). *Utilization of Youth Friendly Services and Associated Factors*.

[28] Bolognesi N., Swartz L. The media management of HIV/AIDS in sub-saharan Africa with particular reference to South Africa: a window to developing communication strategies.

[29] Abebe M., Awoke W. (2014). Utilization of youth reproductive health services and associated factors among high school students in Bahir Dar, Amhara regional state, Ethiopia. *Open Journal of Epidemiology*.

[30] Fikre A. A., Demissie M. (2012). Prevalence of institutional delivery and associated factors in Dodota Woreda (district), Oromia regional state, Ethiopia. *Reproductive Health*.

[31] Shiferaw K., Getahun F., Asres G. (2014). Assessment of adolescents’ communication on sexual and reproductive health matters with parents and associated factors among secondary and preparatory schools’ students in Debremarkos town, North West Ethiopia. *Reproductive Health*.

[32] Kassa M., Abajobir A. A., Gedefaw M. (2014). Level of male involvement and associated factors in family planning services utilization among married men in Debremarkos town, Northwest Ethiopia. *BMC International Health and Human Rights*.

[33] Feleke S. A., Koye D. N., Demssie A. F., Mengesha Z. B. (2013). Reproductive health service utilization and associated factors among adolescents (15–19 years old) in Gondar town, Northwest Ethiopia. *BMC Health Services Research*.

[34] Fawole A. O., Hunyinbo K. I., Adekanle D. A. (2008). Knowledge and utilization of the partograph among obstetric care givers in south west Nigeria. *African Journal of Reproductive Health*.

[35] Ajike S. O., Mbegbu V. C. (2016). Adolescent/youth utilization of reproductive health services: knowledge still a barrier. *Journal of Family Medicine and Health Care*.

[36] Tegegn A., Yazachew M., Gelaw Y. (2008). Reproductive health knowledge and attitude among adolescents: a community based study in Jimma Town, Southwest Ethiopia. *The Ethiopian Journal of Health Development (EJHD)*.

